# Supplementary material for: Lysophosphatidic Acid Receptor 1- and 3-Mediated Hyperalgesia and Hypoalgesia in Diabetic Neuropathic Pain Models in Mice
Source: Cells. 2020 Aug 16;9(8):1906. doi: 10.3390/cells9081906 (PMC7465054; doi:10.3390/cells9081906)
Supplement: Supplementary file 1 [file cells-09-01906-s001.pdf]

cells

MDPI

Supplementary Figures

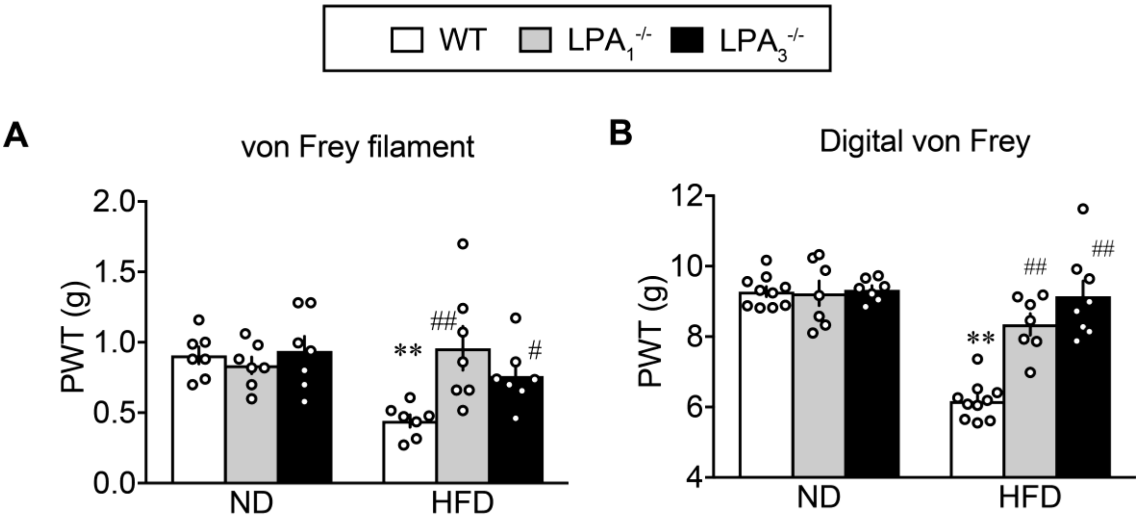

Figure 1. LPA receptor 1 and 3-mediated mechanical allodynia and hyperalgesia in HFD model **(A,B)** Changes in the threshold to show paw withdrawal behaviors in WT, $LPA_1^{-/-}$ and $LPA_3^{-/-}$ mice by von Frey filament test (**A**) and digital con Frey test (**B**). * p <0 .05, ** $p < 0.01$, vs. WT normal diet (ND), two-way ANOVA followed by Bonferroni's multiple comparisons test. # $p < 0.05$, ## $p < 0.01$, vs. WT high fat diet (HFD), two-way ANOVA followed by Tukey's multiple comparisons test (A: n = 7; B: n = 7–10).

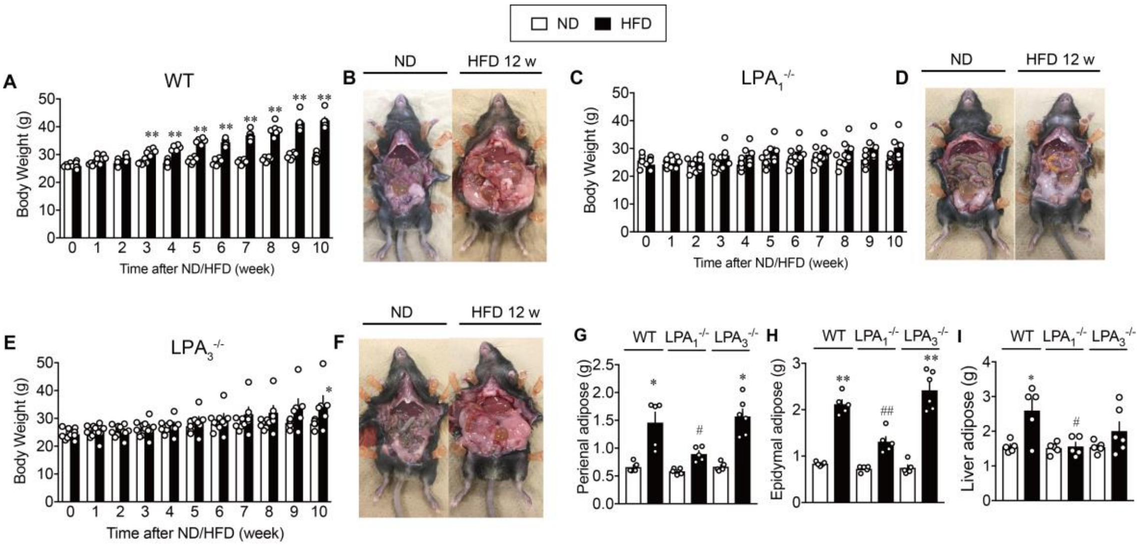

Figure 2. LPA receptor 1 and 3-specific roles in obesity-related changes in HFD model **(A,C,E)** Time-dependent changes in body weight of WT (**A**), $LPA_1^{-/-}$ (**C**) and $LPA_3^{-/-}$ (**E**) mice after the start of ND or HFD feeding throughout 10 weeks. **(B,D,F).** Representative pictures of dissected WT (**B**), $LPA_1^{-/-}$ (**D**) and $LPA_3^{-/-}$ (**F**) mice fed with ND or HFD at 12 weeks. **(G–I)** Changes in the weights of adipose tissues (**G**: perirenal**, H**: epididymal) and liver (**I**) in WT, $LPA_1^{-/-}$ and $LPA_3^{-/-}$ mice. (**A, C, E**) ** $p < 0.01$, vs. ND, two-way ANOVA followed by Bonferroni's multiple comparisons test (n = 6). (**D–F**) ** $p < 0.01$, * $p < 0.05$, vs. ND group at each kind of mouse, ## $p < 0.01$, # $p < 0.05$, vs. WT HFD, one-way ANOVA followed by Tukey's multiple comparisons test (n = 5–6).

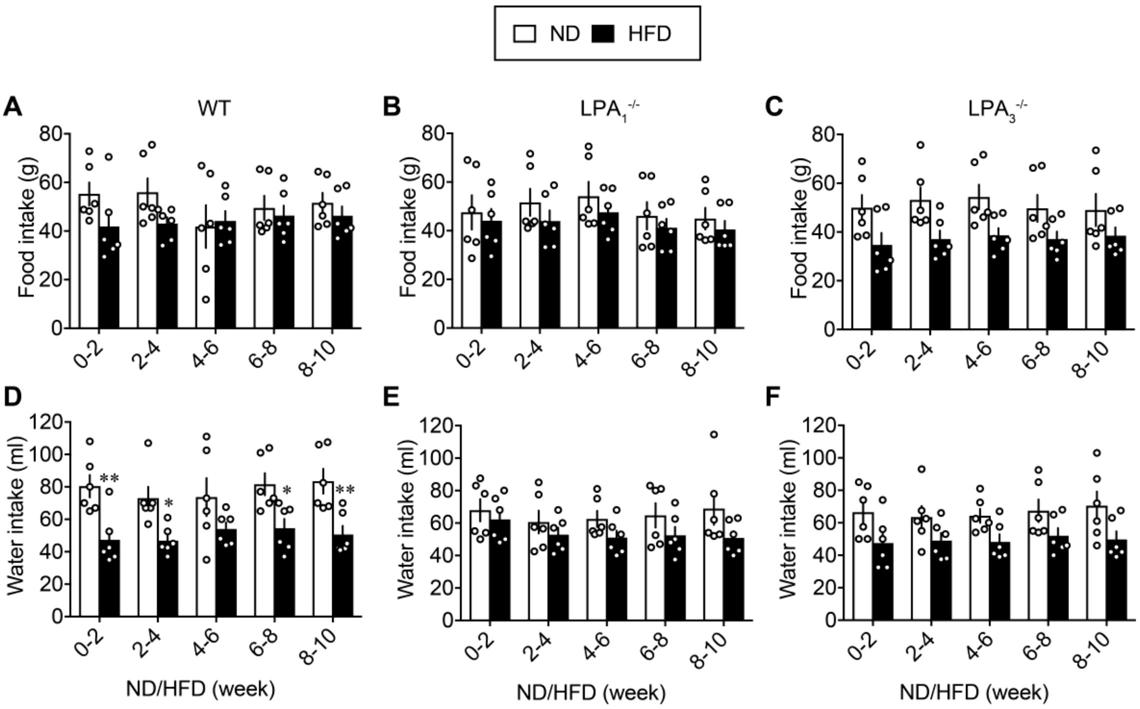

Figure 3. HFD-induced changes in food and water intake in $LPA_1^{-/-}$ and $LPA_3^{-/-}$ mice (A-F) Food intake (**A-C**) and water intake (**D-F**) during indicated periods after the start of ND or HFD feeding in WT (**A, D**), $LPA_1^{-/-}$ (**B, E**) and $LPA_3^{-/-}$ mice (**C, F**). *p<0.05, **p<0.01, vs. Normal diet (ND), two-way ANOVA followed by Bonferroni's multiple comparisons test (n=6).

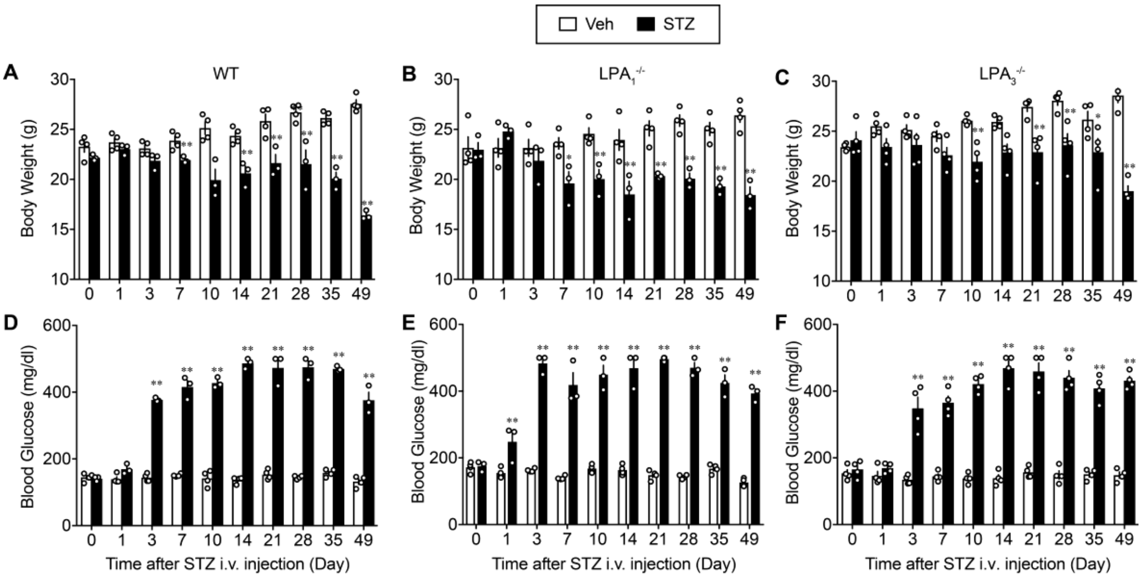

Figure 4. Lack of effects by LPA receptor 1 and 3-deficiency on STZ-induced increase in blood glucose levels and decrease in body weight (A-F) Time-dependent increase in blood glucose levels (A-C) and body weight (D-F) following the vehicle (Veh) or STZ injection (200 mg/kg, i.v.) in WT (A, D), $LPA_1^{-/-}$ (B, E) and $LPA_3^{-/-}$ (C, F) mice after Veh or STZ injection. *p<0.05, **p<0.01, vs. Veh, two-way ANOVA followed by Bonferroni's multiple comparisons test (WT Veh and $LPA_1^{-/-}$ Veh and $LPA_3^{-/-}$ Veh and STZ, n=4; WT STZ and $LPA_1^{-/-}$ STZ, n=3).
